# High bleeding risk in patients undergoing percutaneous coronary intervention with drug-eluting stent implantation: ReCre8 subanalysis

**DOI:** 10.1016/j.ahjo.2022.100227

**Published:** 2022-11-09

**Authors:** Nicole D. van Hemert, Pieter R. Stella, Rik Rozemeijer, Mèra Stein, Peter Frambach, Adriaan O. Kraaijeveld, Saskia Z. Rittersma, Timion A. Meijs, Geert E.H. Leenders, Pim van der Harst, Pierfrancesco Agostoni, Michiel Voskuil, The ReCre8 Study Investigators

**Affiliations:** a Department of Cardiology, University Medical Center Utrecht, Utrecht, the Netherlands; b Department of Cardiology, Zuyderland Medical Center, Heerlen, the Netherlands; c National Institute of Cardiac Surgery and Interventional Cardiology, Luxembourg, Luxembourg; d Department of Cardiology, Hospital Network Antwerp Middelheim, Antwerp, Belgium

**Keywords:** Coronary artery disease, Drug-eluting stent, Percutaneous coronary intervention, High bleeding risk

## Abstract

**Objectives:**

In an all-comers cohort undergoing percutaneous coronary intervention (PCI), we aimed to assess prevalence of high bleeding risk (HBR) patients and impact of HBR and dual antiplatelet therapy (DAPT) on clinical events.

**Background:**

HBR represents a complex subgroup of patients undergoing PCI.

**Methods:**

In the ReCre8 trial, patients undergoing PCI were stratified for troponin status and diabetes and randomized to a permanent polymer zotarolimus-eluting- or polymer-free amphilimus-eluting stent. Patients were treated with 12 months (troponin-positive) or one month (troponin-negative) of DAPT. We evaluated clinical outcomes in patients with and without HBR according to the Academic Research Consortium for High Bleeding Risk criteria.

**Results:**

From a total of 1488 patients included in this subanalysis, 406 patients (27.3 %) were identified as being at HBR. Among HBR patients, target-lesion failure (TLF) was similar after one year yet was higher after three years (13.3 % vs. 9.1 %; p = 0.013), compared to non-HBR patients. There was no difference in Bleeding Academic Research Consortium (BARC) 3 to 5 bleeding, however BARC 2 to 5 bleeding was higher after three years with 4.9 % vs. 3.0 % (p = 0.037). There were no differences between troponin-positive (12-months DAPT) and -negative (1-month DAPT) HBR patients with respect to ischemic and bleeding outcomes.

**Conclusions:**

In this all-comers population of PCI patients, a higher TLF rate among HBR patients at long-term follow-up was found, underlining the complexities involving treatment of HBR patients. We did not observe statistically significant differences in BARC 3 to 5 bleeding between HBR and non-HBR patients regardless of DAPT duration.

**Clinical trial registration:**

URL: http://www.clinicaltrials.gov, unique identifier: NCT02328898.

## Highlights

• Risk factors for high bleeding risk and high ischemic risk overlap.
• Selecting the appropriate treatment in high bleeding risk patients is complicated.
• No difference in three-year major bleeding was found in non-HBR vs. HBR patients.
• A higher ischemic risk was observed with a higher rate of TLF among HBR patients.
• HBR patients require a tailored approach balancing risk of bleeding and ischemia.

## Graphical abstract

**Figure:**
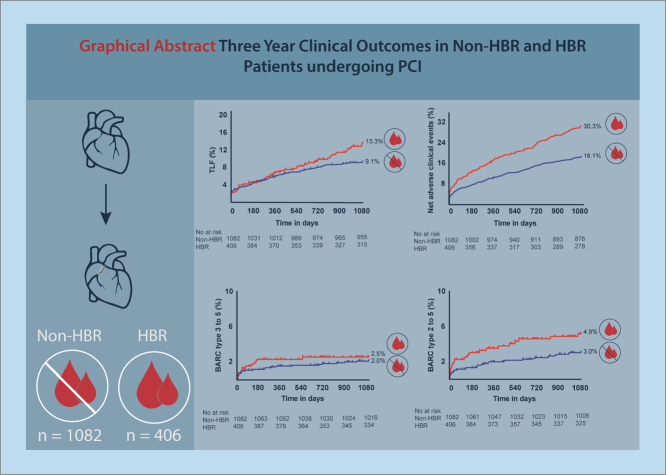

## 1 Introduction

Concerns about late ischemic events after introduction of first-generation drug-eluting stents (DES) induced prolonged dual antiplatelet therapy (DAPT) following percutaneous coronary intervention (PCI) [1]. Despite high bleeding risk (HBR) patients making up 20–40 % of patients undergoing PCI [2], [3], [4], [5], HBR patients have long been neglected in clinical trials as they were considered unsuited to receive the recommended DAPT duration [6]. HBR patients were therefore implanted with bare metal stents (BMS) to facilitate a short DAPT regimen. However in more recent trials, the use of new-generation DES has been shown to be superior to BMS in patients at HBR treated with DAPT for 30 days [7], [8], [9]. Another difficulty was the use of divergent definitions for HBR in clinical trials, withholding further advancements and comparability of study populations [6]. As major bleeding events are associated with increased mortality [10], reducing these complications is of utter importance. It is important to consider is the fact that several risk factors for HBR are shared with indicators of an elevated ischemic risk [11], [12]. This creates additional challenges in finding an optimal treatment regimen for this specific patient population and reinforces the need for patient-tailored therapy.

In this post-hoc subanalysis of the ReCre8 trial [13], we aimed to evaluate the impact of HBR as defined by the Academic Research Consortium for High Bleeding Risk (ARC-HBR) on long-term clinical outcomes in an all-comers population undergoing PCI.

## 2 Methods

### 2.1 Study design and population

The ReCre8 trial [13] is a randomized controlled trial including all-comer patients undergoing PCI. Patients were randomized in a 1:1 ratio to a permanent polymer zotarolimus-eluting stent (PP-ZES; Resolute Integrity, Medtronic Vascular, Santa Rosa, CA) or a polymer-free amphilimus-eluting stent (PF-AES; Cre8, Alvimedica, Istanbul, Turkey) after stratification for troponin status and diabetes. In case of no aspirin and/or Adenosine Diphosphate receptor (ADP) inhibitor use at inclusion, a loading dose was given within seven days prior to the procedure in case of a planned procedure and as soon as possible in case of an emergency procedure. Following PCI, troponin-negative patients were treated with DAPT for one month and troponin-positive patients for 12 months. Troponin-negative patients on oral anticoagulants (OAC) prior to PCI received one month of triple therapy after which they continued with OAC monotherapy. Troponin-positive patients with preceding OAC use received triple therapy for 30 days following PCI, and afterwards continued with clopidogrel and OAC for the remaining 11 months. After one year, patients continued with OAC monotherapy. The study protocol was approved by the institutional review boards of the three participating centers, as well as the Medical Research Ethics Committee Utrecht. All study participants provided written informed consent. All procedures were performed in accordance with institutional guidelines.

### 2.2 HBR definition

Patients in the ReCre8 trial were classified as HBR or non-HBR based on a definition by the ARC-HBR [6]. The ARC-HBR proposed 20 major and minor criteria, categorizing patients into patients at HBR and patients not at HBR. Table S1 provides an overview of the major and minor criteria proposed by the ARC-HBR and the criteria used in this analysis. The variable ‘anticipated use of long-term oral anticoagulants’ was adapted to (N)OAC use at inclusion. Several variables initially were not collected in our data set and were therefore retrospectively collected in the share of patients included in the University Medical Center Utrecht. Multiple imputation was then performed to account for missing data. One of the criteria – non-deferrable major surgery on DAPT – was an exclusion criterion in the ReCre8 trial (planned surgery within three months) and was not included in this analysis. As proposed by the ARC-HBR, patients with at least one major or two minor criteria were classified as HBR.

### 2.3 Study endpoints

The primary endpoint of the ReCre8 trial was target-lesion failure (TLF), a composite of cardiac death, target-vessel myocardial infarction and target-lesion revascularization. The secondary endpoint of net adverse clinical events (NACE) was composed of all-cause death, any myocardial infarction, any unplanned revascularization, stroke, and Bleeding Academic Research Consortium (BARC) type 3 to 5 bleeding. Additionally, all endpoint components and BARC type 2 to 5 bleeding were assessed separately. Comprehensive endpoint definitions have been previously described [14] and were defined according to definitions by the Academic Research Consortium [15], [16]. All endpoints were prospectively collected with active follow-up and were adjudicated by the Clinical Event Committee.

### 2.4 Statistical analysis

Baseline characteristics are reported as counts and percentages (binomial variables) or mean ± standard deviation (continuous variables). Clinical endpoints were assessed by Kaplan-Meier time-to-event estimates and compared using log-rank test. Time-to-event was defined as the number of days between inclusion and first occurrence of any (component of the composite) endpoint. In a supplemental analysis, endpoints were assessed during DAPT and during aspirin or OAC monotherapy until final follow-up at three years. Follow-up was censored at 1095 days. Differences were considered statistically significant at a 2-tailed p-value of 0.05. As retrospective data collection on HBR criteria was only feasible in patients included in the UMC Utrecht, multiple data imputation was used to account for missing data to minimize bias. Imputation was performed for all HBR criteria, as well as scarce missing values in prospectively collected baseline variables. Fully conditional specification multiple imputation was performed by means logistic regression for categorical variables and predictive mean matching for continuous variables. Based on the percentage of missing variables across patients [17], 41 datasets were created. The presence of HBR as well as Kaplan-Meier time-to-event estimates were assessed in all imputed datasets and log-rank values were subsequently pooled. Counts, percentages and survival curves were generated using an averaged dataset and Chi-Square tests were pooled. Statistical analyses were performed using SAS version 9.4 (SAS Institute, Cary, NC), SPSS statistics version 26 (IBM, New York, NY) and RStudio version 1.3.1093 (RStudio, Boston, MA).

## 3 Results

### 3.1 Baseline and procedural characteristics

From the 1491 patients included in the ReCre8 trial, 1488 patients were included in the current analysis after three consent withdrawals. A total of 406 patients (27.3 %) were identified as HBR. Baseline characteristics are shown in Table 1. In the HBR population, 208 patients (51.2 %) were over 75 years of age, 123 patients (30.3 %) used (N)OAC therapy, and anemia was present in 147 HBR patients (36.2 %). A total of 187 patients (46.1 %) in the HBR population had CKD; 22 patients (5.4 %) had severe CKD and 165 patients (40.6 %) had moderate CKD. Preprocedural ADP receptor inhibitor therapy is shown in Table S2. Baseline characteristics in HBR patients stratified for troponin status are shown in Table S3. Antiplatelet- and anticoagulation therapy at 12 months is shown in Table S4.

**Table 1:** Baseline characteristics.

	Overall (n = 1488)	Non-HBR (n = 1082)	HBR (n = 406)	p value
Clinical characteristics				
Age ≥ 75 years	303 (20.4)	95 (8.8)	208 (51.2)	**<0.001**
Male sex	1139 (76.5)	851 (78.7)	288 (70.9)	**0.002**
Body mass index (kg/m^2^)	27.3 ± 4.42	27.4 ± 4.22	27.1 ± 4.94	0.34
Hypertension	821 (55.2)	535 (49.4)	286 (70.4)	**<0.001**
Diabetes mellitus	303 (20.4)	179 (16.5)	124 (30.5)	**<0.001**
Current smoker	384 (25.8)	315 (29.1)	69 (17.0)	**<0.001**
Family history of cardiovascular disease	565 (38.0)	453 (41.9)	112 (27.6)	**<0.001**
OAC use	85 (5.7)	0 (0.0)	85 (20.9)	**<0.001**
NOAC use	38 (2.6)	0 (0.0)	38 (9.4)	**<0.001**
Major surgery/trauma within 30 days before PCI	30 (2.0)	0 (0.0)	30 (7.4)	**0.049**
Laboratory values				
Moderate CKD	200 (13.4)	35 (3.2)	165 (40.6)	**<0.001**
Severe or end-stage CKD	22 (1.5)	0 (0.0)	22 (5.4)	**<0.001**
Thrombocytopenia	14 (0.9)	0 (0.0)	14 (3.4)	**<0.001**
Hemoglobin < 11 g/dL	48 (3.2)	0 (0.0)	48 (11.8)	**<0.001**
Hemoglobin 11–12.9 g/dL (♂) or 11–11.9 g/dL (♀)	169 (11.4)	70 (6.5)	99 (24.4)	**<0.001**
Relevant medical history				
Previous MI	297 (20.0)	178 (16.5)	119 (29.3)	**<0.001**
Previous PCI	303 (20.4)	199 (18.4)	104 (25.6)	**<0.001**
Previous CABG	138 (9.3)	69 (6.4)	69 (17.0)	**<0.001**
Liver cirrhosis with portal hypertension	33 (2.2)	0 (0.0)	33 (8.1)	**0.048**
Prior bleeding	55 (3.7)	0 (0.0)	55 (13.5)	**0.003**
Previous spontaneous or traumatic ICH	33 (2.2)	0 (0.0)	33 (8.1)	**0.016**
Active malignancy	40 (2.7)	0 (0.0)	40 (9.9)	**<0.001**
Previous ischemic stroke	44 (3.0)	12 (1.1)	32 (7.9)	**<0.001**
Stent type				0.80
Resolute Integrity	741 (49.8)	541 (50.0)	200 (49.3)	
Cre8	747 (50.2)	541 (50.0)	206 (50.7)	
Clinical presentation				**0.003**
Troponin-negative	889 (59.7)	621 (57.4)	268 (66.0)	
Troponin-positive	599 (40.3)	461 (42.6)	138 (34.0)	
Number of diseased coronary vessels				**<0.001**
1	837 (56.2)	645 (59.6)	192 (47.3)	
2	424 (28.5)	294 (27.2)	130 (32.0)	
≥3	227 (15.3)	143 (13.2)	84 (20.7)	

Data are n (%) or mean ± standard deviation. Abbreviations: CABG, coronary artery bypass grafting; CKD, chronic kidney disease; ICH, intracranial hemorrhage; MI, myocardial infarction; (N)OAC, (new) oral anticoagulation; PCI, percutaneous coronary intervention. A p value in bold indicates a statistically significant difference (p < 0.05).

### 3.2 Clinical outcomes

#### 3.2.1 Non-HBR vs. HBR patients

**Fig. 1:**
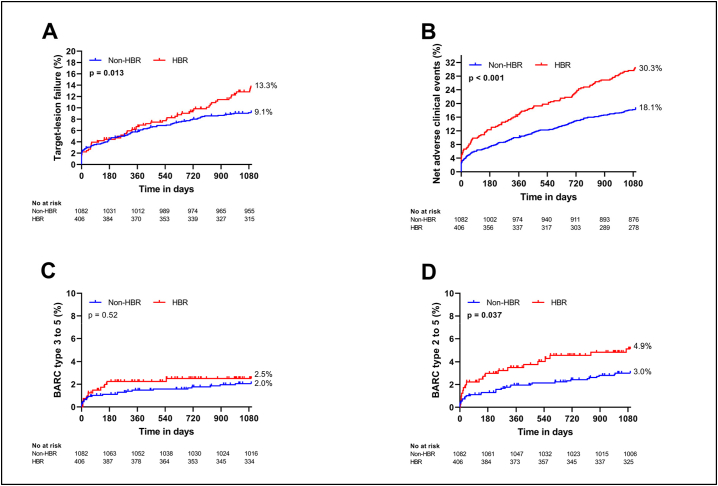
Kaplan-Meier curves for the primary endpoint of TLF, NACE and bleeding events after three years in patients with and without HBR. Abbreviations: BARC, Bleeding Academic Research Consortium; HBR, high bleeding risk; NACE, net adverse clinical events; TLF, target-lesion failure.

Clinical outcomes for non-HBR and HBR patients are shown in Table 2 and Kaplan-Meier analyses over three years are shown in Fig. 1. Between index PCI and three years, TLF occurred more frequently among HBR patients with 9.1 % vs. 13.3 % (p = 0.013). Similarly, NACE occurred in 196 non-HBR patients (18.1 %) and 123 HBR patients (30.3 %; p < 0.001). There was no statistically significant difference in BARC type 3 to 5 bleeding (22 non-HBR patients (2.0 %) vs. 10 HBR patients (2.5 %) (p = 0.52)). After three years, the endpoints all-cause death, cardiac death, stroke and BARC type 2 to 5 bleeding were higher among HBR patients.

**Table 2:** Clinical events in HBR vs. non-HBR patients.

	0–1 year				0–3 years			
	Overall (n = 1488)	Non-HBR (n = 1082)	HBR (n = 406)	p value	Overall (n = 1488)	Non-HBR (n = 1082)	HBR (n = 406)	p value
TLF	88 (5.9)	63 (5.8)	25 (6.2)	0.64	152 (10.2)	98 (9.1)	54 (13.3)	**0.013**
NACE	176 (11.8)	109 (10.1)	67 (16.5)	**<0.001**	319 (21.4)	196 (18.1)	123 (30.3)	**<0.001**
All-cause death	35 (2.4)	16 (1.5)	19 (4.7)	**<0.001**	96 (6.5)	38 (3.5)	58 (14.3)	**<0.001**
Cardiac death	20 (1.3)	9 (0.8)	11 (2.7)	**0.010**	45 (3.0)	20 (1.8)	25 (6.2)	**<0.001**
Myocardial infarction	53 (3.6)	33 (3.0)	20 (4.9)	0.059	82 (5.5)	52 (4.8)	30 (7.4)	0.052
TV-MI	35 (2.4)	28 (2.6)	7 (1.7)	0.44	48 (3.2)	37 (3.4)	11 (2.7)	0.54
Stent thrombosis	15 (1.0)	8 (0.7)	7 (1.7)	0.074	16 (1.1)	9 (0.8)	7 (1.7)	0.11
Any unplanned revascularization	72 (4.8)	54 (5.0)	18 (4.4)	0.64	161 (10.8)	115 (10.6)	46 (11.3)	0.70
TLR	42 (2.8)	33 (3.0)	9 (2.2)	0.51	83 (5.6)	60 (5.5)	23 (5.7)	0.69
Stroke	12 (0.8)	6 (0.6)	6 (1.5)	0.073	27 (1.8)	15 (1.4)	12 (3.0)	**0.025**
BARC 3 to 5	25 (1.7)	16 (1.5)	9 (2.2)	0.28	32 (2.2)	22 (2.0)	10 (2.5)	0.52
BARC 2 to 5	35 (2.4)	21 (1.9)	14 (3.4)	0.061	52 (3.5)	32 (3.0)	20 (4.9)	**0.037**

Data are n (%). Abbreviations: BARC, Bleeding Academic Research Consortium; HBR, high bleeding risk; NACE, net adverse clinical events; TLF, target-lesion failure; TLR, target-lesion revascularization; TV-MI; target-vessel myocardial infarction. A p value in bold indicates a statistically significant difference (p < 0.05).

Results between index PCI and one year are shown in Table 2 and Fig. S1. TLF occurred in 63 non-HBR patients (5.8 %) vs. 27 HBR patients (6.7 %; p = 0.48). NACE occurred in 109 non-HBR patients (10.1 %) vs. 70 HBR patients (17.2 %; p < 0.001). Neither BARC type 3 to 5 bleeding (16 patients (1.5 %) vs. nine patients (2.2 %; p = 0.28)), nor BARC type 2 to 5 bleeding (21 patients (1.9 %) vs. 14 patients (3.4 %); p = 0.061) were statistically significantly higher among HBR patients. Regarding separate endpoint components, all-cause death, cardiac death, and stroke were higher among HBR patients in the first year following PCI.

#### 3.2.2 Troponin-negative (one month DAPT) vs. troponin-positive (12 months DAPT) HBR patients

**Fig. 2:**
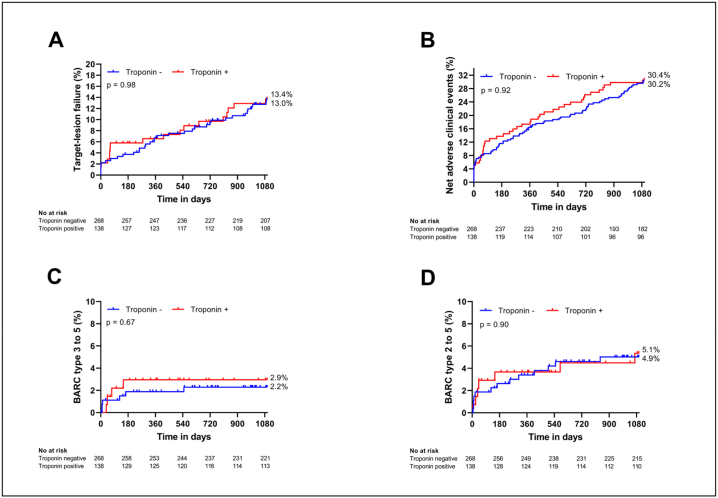
Kaplan-Meier curves for clinical outcomes at three years among high bleeding risk patients treated with one month (troponin-negative) vs. twelve months (troponin-positive) of dual antiplatelet therapy. Abbreviations: BARC, Bleeding Academic Research Consortium.

Clinical outcomes in troponin-negative and troponin-positive patients for the subgroup of HBR patients are shown in Table S5 and Fig. 2. After three years, TLF occurred in 36 troponin-negative patients (13.4 %) and 18 troponin-positive patients (13.0 %; p = 0.98). NACE was similar between groups, occurring in 81 troponin-negative patients (30.2 %) vs. 42 troponin-positive patients (30.4 %; p = 0.92). There were no differences in any separate endpoint components. BARC type 3 to 5 bleeding occurred in six troponin-negative patients (2.2 %) vs. four troponin-positive patients (2.9 %; p = 0.67). BARC type 2 to 5 bleeding occurred in 13 troponin-negative patients (4.9 %) vs. seven troponin-positive patients (5.1 %; p = 0.90). Outcomes between index PCI and one year showed comparable rates for all evaluated endpoints between the troponin-negative and -positive population. Data on the troponin-negative and –positive HBR and non-HBR populations are shown in Tables S6 and S7 and Figs. S2 and S3.

#### 3.2.3 HBR patients implanted with the PP-ZES vs. PF-AES

Clinical outcomes in HBR patients in the two stent arms are shown in Table S8 and clinical outcomes in non-HBR patients in the two stent arms are shown in Table S9. Between index PCI and one year follow-up, there were no differences between any of the evaluated endpoints. TLF occurred in 6.5 % of PP-ZES patients vs. 5.8 % of PF-AES patients (p = 0.77). After three years follow-up, TLF occurred in 14 % of PP-ZES HBR patients and in 12.6 % of PF-AES HBR patients (p = 0.64). NACE occurred in 33 % vs. 27.7 % (p = 0.26). There were no differences in the separate endpoint components apart from a higher rate of any unplanned revascularization in the PP-ZES arm after three years (14.5 % vs. 8.3 %; p = 0.042). The rates for BARC type 3 to 5 and BARC type 2 to 5 bleeding were not statistically significantly different although BARC type 2 to 5 bleeding showed a clear trend towards more events in the PP-ZES arm (7.0 % vs. 2.9 %; p = 0.054). Among non-HBR patients, there were no differences between the two stent arms in any endpoints.

## 4 Discussion

The main findings of this subanalysis are 1) a comparable rate of TLF after one year yet a higher rate of TLF among HBR patients after three years, 2) a higher NACE rate after both one and three years, and 3) no statistically significant differences in BARC type 3 to 5 between non-HBR and HBR patients. Regarding the analysis on troponin-negative (short DAPT) vs. troponin-positive (12 months DAPT) HBR patients, no differences in clinical outcomes were observed.

In the ARC-HBR definition, HBR was defined as a risk of BARC type 3 or 5 bleeding of ≥4 % one year after PCI or a risk of ≥1 % of intracranial hemorrhage after one year – which was not an endpoint in the ReCre8 trial. These rates were chosen from former trials on HBR patients. The rate of BARC type 3 or 5 bleeding of 4 % after one year was not reached in our HBR population: the combined rate of BARC type 3 to 5 bleeding among all HBR patients was 2.5 %. A possible explanation for these lower rates may be the short DAPT duration (i.e. one month) in a major share (66 %) of our HBR patients. Additionally, in the ReCre8 trial a transradial approach for PCI was used in 89 % of our HBR patients. The use of a transradial approach has been shown to reduce hemorrhagic events [18] and may therefore have positively altered the bleeding rates in our population. The exclusion of patients with planned major surgery in the ReCre8 trial may have also contributed to lower bleeding rates.

Several other trials have performed a validation of the ARC-HBR criteria in their study population. In a validation analysis in a Northern-American cohort [5], bleeding occurred in 9.1 % of HBR patients after one year with a higher rate of both peri-procedural and post-discharge bleeding among HBR patients. Secondary endpoints all-cause mortality (4.7 %) and post-discharge myocardial infarction (4.2 %) were also higher among HBR patients after one year. As compared to this trial, the ReCre8 trial had a substantially higher rate of transradial approaches. This may have led to the higher rate of major bleeding, with periprocedural and in-hospital bleeding occurring in 4.8 % of HBR patients. Additionally, a different definition for the bleeding endpoint was used in this trial. Regarding secondary endpoints, all-cause mortality after one year was comparable with the 4.7 % in the ReCre8 trial. Any myocardial infarction occurred in 4.9 % of patients in the ReCre8 trial – including periprocedural and in-hospital events. Inclusion of in-hospital events presumably led to a marginally higher rate of myocardial infarction in our analyses. Unfortunately, the potential impact of DAPT duration cannot be weighed as this was neither dictated nor registered in the validation cohort [5].

The clinical outcomes after one year in our population differed from other study populations treated with one month of DAPT. In the Onyx One Clear trial [2], outcomes between one month and one year were evaluated in patients who were DAPT compliant and event-free after one month. TLF occurred in 8.1 % of patients, as compared to 6.7 % of the troponin-negative HBR patients in the ReCre8 trial including events in the first month. Target-lesion revascularization was also higher: 3.4 % in Onyx One Clear vs. 2.6 % in the ReCre8 trial, with exclusion of patients with an event in the first month in Onyx One Clear. Interestingly, stent thrombosis was higher in the ReCre8 trial with 1.9 % in the first year (n = 5 with 1 case occurring in the first month), as compared to 0.7 % between one month and one year. Although the share of patients with diabetes and complex lesion anatomy [19] was higher in the Onyx One Clear trial, a longer total stent length and more stents per patient were implanted in the ReCre8 trial. Additionally, 17 % of HBR patients in the ReCre8 trial presented with a STEMI as compared to 4 % of patients in Onyx One Clear. Furthermore, as the Onyx Resolute stent implanted in Onyx One Clear is the successor of the Resolute Integrity stent, its enhanced characteristics may have contributed to the lower rate of stent thrombosis. It should however also be acknowledged that patients with an ischemic or bleeding event in the first month following PCI were excluded from the Onyx One Clear trial, which presumably precluded high-risk patients.

Regarding bleeding endpoints, both BARC type 3 to 5 bleeding (4.0 % vs. 2.9 %) and BARC type 2 to 5 bleeding (11.7 % vs. 3.6 %) were considerably lower in the ReCre8 trial. Both trials included a complex patient population with high incidences of risk factors. The lower rates of bleeding in this analysis may in part be caused by a different definition of HBR. Additionally, in the ReCre8 trial 89 % of the HBR patients were treated transradial as compared to 66 % in Onyx One Clear.

The supposedly opposing risks of bleeding and ischemia coincide in HBR patients, as risk factors for HBR and high ischemic risk overlap – a phenomenon clearly visible in our HBR population. Even during DAPT, troponin-positive HBR patients showed higher rates of NACE, all-cause- and cardiac death and stroke as compared to non-HBR patients, in the absence of significantly higher bleeding rates. These data suggest that HBR patients as identified by the ARC-HBR criteria form a complex and more diseased population in general. This can also be seen from differences in baseline risks, not all of which are inherent to the ARC-HBR division. Aside from implicit higher rates of CKD and higher age, HBR patients more frequently have hypertension, diabetes, prior revascularization, left main- and multivessel disease. Finding a balance between these two opposing risks and adapting the treatment strategy for the individual risk in each patient is important in minimizing adverse events. In an effort to do so, the ARC-HBR developed a trade-off model [11] aiding physicians in this challenge. It should be acknowledged that although the three-year TLF rate was higher among HBR patients, this was driven by a higher rate of cardiac death and was not attributable to the treated culprit lesion. This could be a reflection of a more diseased population in the cardiovascular system as a whole, rather than an after-effect of the procedure.

Although several validation papers have been published regarding the ARC-HBR criteria, most publications to date have reported on one-year follow up and few cohorts have been analyzed on very long-term follow-up in comparable patient populations. An analysis on long-term follow-up of HBR patients based on the ARC-HBR criteria has been performed in a retrospective single-center study [20]. After eight years, BARC 3 or 5 bleeding occurred in 5.7 % of non-HBR patients vs. 16.2 % of HBR patients (p < 0.0001). The primary ischemic endpoint – a composite of myocardial infarction, stent thrombosis and ischemic stroke – was significantly higher in the HBR population with 8.3 % vs. 12.7 % (p = 0.001). Both all-cause death (10.4 % vs. 42.7 %) and cardiac death (3.6 % vs. 16.6 %) were significantly higher in the HBR population, leading to relative hazard ratios exceeding five. These long-term data stress the importance of focusing on this important subpopulation in future research.

To assess similarities between the ARC-HBR definition and a previous bleeding score, we compared this classification to PRECISE-DAPT scores for ReCre8 patients [21]. In 1036 patients (69.6 %), the presence of HBR based on the ARC-HBR criteria and PRECISE-DAPT matched. In 59 cases (4.0 %), patients were classified as not being at HBR in this analysis yet PRECISE-DAPT score indicated HBR. Conversely, among patients classified as HBR in this analysis, 81 patients (5.4 %) were assigned to be at moderate risk of bleeding based on PRECISE-DAPT scores, and 131 patients (8.8 %) were classified as being at low or very low risk of bleeding. In PRECISE-DAPT [22], four criteria are similar to the criteria proposed by the ARC-HBR but in a point-based scoring system rather than binary (aside from previous bleed) and with different cut-offs. These definitions discriminate between patients with and without HBR in a similar manner in almost 70 % of patients. The PRECISE-DAPT score appears to be less likely to identify a patient as being at HBR with 253 HBR patients in this cohort based on PRECISE-DAPT scores vs. 406 based on the ARC-HBR definition.

### 4.1 Limitations

There are several limitations that should be acknowledged. First, not all risk factors for HBR were prospectively collected. To minimize information bias, several variables were collected retrospectively for patients included in the University Medical Center Utrecht – 75 % of included patients – and imputation was performed to account for unavailable data. However as some ARC-HBR criteria were not present in the population for which retrospective data collection was performed, no imputation was possible. Remaining missing variables may have caused an underestimation of the share of HBR patients and accompanying risks, as the defined 4 % BARC 3 to 5 bleeding was not reached in our HBR population. Second, one of the criteria for HBR as described by the ARC-HBR was an exclusion criterion in the ReCre8 trial which may have prevented inclusion of certain HBR patients. Third, the choice of DAPT duration in the ReCre8 trial was dictated by clinical presentation rather than by randomization. It is important to consider this analysis as exploratory as these subgroups are not directly comparable. Last, this post-hoc subanalysis of the ReCre8 trial was not powered for subgroup analyses nor was it powered or designed for evaluation of clinical events with low incidences such as bleeding. The results from this subanalysis should therefore be considered hypothesis-generating.

## 5 Conclusions

In this cohort of all-comer PCI patients implanted with new-generation DES, comparable rates of BARC type 3 to 5 bleeding were observed between HBR and non-HBR patients regardless of clinical presentation and its corresponding DAPT duration. The complexities involving treatment of HBR patients is reflected by the higher TLF rate among HBR patients at long-term follow-up in the absence of thrombotic protection in the form of DAPT.

## Sources of funding

The ReCre8 trial was funded by the three participating centers.

## CRediT authorship contribution statement

**Nicole D. van Hemert:** Writing – review & editing, Writing – original draft, Visualization, Formal analysis, Investigation. **Pieter R. Stella:** Conceptualization, Methodology, Writing – review & editing, Supervision. **Rik Rozemeijer:** Writing – review & editing, Formal analysis, Investigation. **Mèra Stein:** Conceptualization, Methodology, Writing – review & editing. **Peter Frambach:** Conceptualization, Methodology, Writing – review & editing. **Adriaan O. Kraaijeveld:** Conceptualization, Methodology, Writing – review & editing. **Saskia Z. Rittersma:** Conceptualization, Methodology, Writing – review & editing. **Timion A. Meijs:** Writing – review & editing, Formal analysis, Investigation. **Geert E.H. Leenders:** Conceptualization, Methodology, Writing – review & editing. **Pim van der Harst:** Writing – review & editing, Supervision. **Pierfrancesco Agostoni:** Conceptualization, Methodology, Writing – review & editing. **Michiel Voskuil:** Conceptualization, Methodology, Writing – review & editing, Supervision.

## Declaration of competing interest

The authors declare that they have no known competing financial interests or personal relationships that could have appeared to influence the work reported in this paper.

## Appendix A Supplementary data

Supplementary material Image 1
