# Supplementary material for: High bleeding risk in patients undergoing percutaneous coronary intervention with drug-eluting stent implantation: ReCre8 subanalysis
Source: Am Heart J Plus. 2022 Nov 9;24:100227. doi: 10.1016/j.ahjo.2022.100227 (PMC10978429; doi:10.1016/j.ahjo.2022.100227)
Supplement: Supplementary file 1 — Supplementary material [file mmc1.docx]

**Supplemental data**

| Table S1. Adapted ARC-HBR Criteria in ReCre8 | | |  |
| --- | --- | --- | --- |
| Major criteria | **Minor criteria** | **Adaptation ReCre8** | **% missing** |
|  | Age ≥ 75y | Unadapted | 0.0 |
| Anticipated use of long-term oral anticoagulation |  | (N)OAC use | 4.6 |
| Severe or end-stage CKD | Moderate CKD | Unadapted | 3.8 |
| Hemoglobin < 11g/dL | Hemoglobin 11-12.9 g/dL (♂) or 11-11.9 g/dL (♀) | Unadapted | 4.5 |
| Spontaneous bleeding^*^ in the past six months or at any time if recurrent | Spontaneous bleeding^*^ within past 12 months | Unadapted^‡^ | 24.5 |
| Moderate or severe baseline thrombocytopenia |  | Unadapted | 31.0 |
| Chronic bleeding diathesis |  | Unadapted^‡^ | 24.5 |
| Liver cirrhosis with portal hypertension |  | Unadapted^‡^ | 24.5 |
|  | Long-term use of oral NSAIDs or steroids | Not available | N/A |
| Active malignancy^†^ within past 12 months |  | Unadapted^‡^ | 24.5 |
| Previous spontaneous ICH, previous traumatic ICH within past 12 months, presence of bAVM, moderate/severe ischemic stroke within past six months |  | Unadapted^‡^ | 24.5 |
| Nondeferrable major surgery on DAPT |  | Planned surgery within three months excluded | N/A |
| Major surgery/trauma within 30 days before PCI |  | Unadapted^‡^ | 24.5 |

Abbreviations: ARC-HBR, Academic Research Consortium for High Bleeding Risk; bAVM, brain Arteriovenous Malformation; CKD, Chronic Kidney Disease; DAPT, Dual Antiplatelet Therapy; NSAIDs, Non Steroidal Anti-Inflammatory Drug; PCI, Percutaneous Coronary Intervention. ^*^ requiring hospitalization or transfusion. ^†^ excluding non-melanome skin cancer. ^‡^ retrospectively collected from available data in electronic patient file in one of the participating centers (n=1124).

| Table S2. Pre-procedural ADP receptor inhibitor therapy | | | | |
| --- | --- | --- | --- | --- |
|  | Overall (n=1488) | Non-HBR (n=1082) | HBR (n=406) | p-value |
| Preprocedural |  |  |  |  |
| Clopidogrel loading or q.d. | 593 (39.9) | 401 (37.1) | 192 (47.3) | **0.001** |
| Ticagrelor loading or q.d. | 548 (36.8) | 461 (42.6) | 87 (21.4) | **<0.001** |
| Prasugrel loading or q.d. | 14 (0.9) | 9 (0.8) | 5 (1.2) | 0.50 |

Data are counts and percentages or median and inter-quartile range. Abbreviations: ADP, Adenosine Diphosphate receptor inhibitor; HBR, High Bleeding Risk; q.d., quaque die.

| Table S3. Baseline characteristics in HBR patients stratified for troponin status | | | |  |
| --- | --- | --- | --- | --- |
|  | Overall (n=406) | Troponin positive (n=138) | Troponin negative (n=268) | p-value |
| Clinical characteristics |  |  |  |  |
| Age ≥ 75y | 208 (51.2) | 78 (56.5) | 130 (48.5) | 0.13 |
| Male sex | 288 (70.9) | 100 (72.5) | 188 (70.1) | 0.63 |
| Body mass index (kg/m^2^) | 27.1 ± 4.94 | 26.3 ± 4.39 | 27.6 ± 5.14 | **0.007** |
| Hypertension | 286 (70.4) | 94 (68.1) | 192 (71.6) | 0.76 |
| Diabetes Mellitus | 124 (30.5) | 41 (29.7) | 83 (31.0) | 0.79 |
| Current smoker | 69 (17.0) | 25 (18.1) | 44 (16.4) | 0.67 |
| Family history of cardiovascular disease | 112 (27.6) | 30 (21.7) | 82 (30.6) | 0.17 |
| (N)OAC use | 123 (30.3) | 25 (18.1) | 98 (36.6) | **<0.001** |
| Major surgery/trauma within 30 days before PCI | 30 (7.4) | 5 (3.6) | 25 (9.3) | **0.044** |
| Laboratory values |  |  |  |  |
| Moderate CKD | 165 (40.6) | 73 (52.9) | 92 (34.3) | **<0.001** |
| Severe or end-stage CKD | 22 (5.4) | 8 (5.8) | 14 (5.2) | 0.81 |
| Thrombocytopenia | 14 (3.4) | 8 (5.8) | 6 (2.2) | 0.063 |
| Hemoglobin < 11g/dL | 48 (11.8) | 27 (19.6) | 21 (7.8) | **0.001** |
| Hemoglobin 11-12.9 g/dL (♂) or 11-11.9 g/dL (♀) | 99 (24.4) | 39 (28.3) | 60 (22.4) | 0.19 |
| Relevant medical history |  |  |  |  |
| Previous MI | 119 (29.3) | 29 (21.0) | 90 (33.6) | **0.031** |
| Previous PCI | 104 (25.6) | 24 (17.4) | 80 (29.9) | **0.006** |
| Previous CABG | 69 (17.0) | 18 (13.0) | 51 (19.0) | 0.13 |
| Liver cirrhosis with portal hypertension | 33 (8.1) | 10 (7.2) | 23 (8.9) | 0.64 |
| Prior bleeding | 55 (13.5) | 9 (6.5) | 46 (17.2) | **0.003** |
| Previous spontaneous or traumatic ICH | 33 (8.1) | 3 (2.2) | 30 (11.2) | **0.002** |
| Active malignancy | 40 (9.9) | 10 (7.2) | 30 (11.2) | 0.21 |
| Previous ischemic stroke | 32 (7.9) | 7 (5.1) | 25 (9.3) | 0.13 |
| Stent type |  |  |  | 0.14 |
| Resolute Integrity | 200 (49.3) | 75 (54.3) | 125 (46.6) |  |
| Cre8 | 206 (50.7) | 63 (45.7) | 143 (53.4) |  |
| Number of diseased coronary vessels |  |  |  | 0.48 |
| 1 | 192 (47.3) | 60 (43.5) | 132 (49.3) |  |
| 2 | 130 (32.0) | 42 (30.4) | 88 (32.8) |  |
| ≥ 3 | 84 (20.7) | 36 (26.1) | 48 (17.9) |  |

Data are n (%) or mean ± standard deviation. Abbreviations: CABG, Coronary Artery Bypass Grafting; CKD, Chronic Kidney Disease; ICH, Intracranial Haemorrhage; MI, Myocardial Infarction; (N)OAC, (new) Oral Anticoagulation; PCI, Percutaneous Coronary Intervention.

| Table S4. Antiplatelet- and anticoagulation therapy at 12 months | | | | | | | | |
| --- | --- | --- | --- | --- | --- | --- | --- | --- |
|  | Overall  (n=1453) | | Non-HBR  (n=1066) | | | HBR  (n=387) | | |
|  | Troponin positive (n=585) | Troponin negative (n=868) | | Troponin positive (n=457) | Troponin  Negative (n=609) | | Troponin positive (n=128) | Troponin negative (n=259) |
| Aspirin | 535 (91.5) | 757 (87.2) | | 432 (94.5) | 577 (94.8) | | 103 (80.5) | 180 (69.5) |
| ADP-inhibitor | 213 (36.4) | 133 (15.3) | | 166 (36.3) | 92 (15.1) | | 47 (36.7) | 41 (15.8) |
| NOAC | 9 (1.5) | 32 (3.7) | | 3 (0.7) | 7 (1.2) | | 6 (4.7) | 25 (9.7) |
| OAC | 40 (6.8) | 78 (9.0) | | 18 (3.9) | 18 (3.0) | | 22 (17.2) | 60 (23.2) |

Data are n (%). Abbreviations: ADP, Adenosine Diphosphate; HBR, High Bleeding Risk; NOAC, New Oral Anticoagulation; OAC, Oral Anticoagulation.

| Table S5. Clinical events in troponin negative vs. troponin positive HBR patients | | | | | | |  | |  | |  |
| --- | --- | --- | --- | --- | --- | --- | --- | --- | --- | --- | --- |
|  | 0 – 1 year | | | |  | 0 – 3 years | | | | | |
|  | Overall (n=406) | Troponin - (n=268) | Troponin + (n=138) | p value | Overall (n=406) | Troponin –(n=268) | | Troponin + (n=138) | | p value | |
| TLF | 25 (6.2) | 17 (6.7) | 8 (5.8) | 0.86 | 54 (13.3) | 36 (13.4) | | 18 (13.0) | | 0.98 | |
| NACE | 67 (16.5) | 43 (16.0) | 24 (17.4) | 0.72 | 123 (30.3) | 81 (30.2) | | 42 (30.4) | | 0.92 | |
| All-cause death | 19 (4.7) | 9 (3.4) | 10 (7.2) | 0.078 | 58 (14.3) | 36 (13.4) | | 22 (15.9) | | 0.47 | |
| Cardiac death | 11 (2.7) | 7 (2.6) | 4 (2.9) | 0.85 | 25 (6.2) | 15 (5.6) | | 10 (7.2) | | 0.49 | |
| Myocardial infarction | 20 (4.9) | 15 (5.6) | 5 (3.6) | 0.39 | 30 (7.4) | 20 (7.5) | | 10 (7.2) | | 0.97 | |
| TV-MI | 7 (1.7) | 5 (1.9) | 2 (1.4) | 0.76 | 11 (2.7) | 5 (1.9) | | 6 (4.3) | | 0.14 | |
| Stent thrombosis^*^ | 7 (1.7) | 5 (1.9) | 2 (1.4) | 0.78 | 7 (1.7) | 5 (1.9) | | 2 (1.4) | | 0.78 | |
| Any unplanned revascularization | 18 (4.4) | 14 (5.2) | 4 (2.9) | 0.31 | 46 (11.3) | 34 (12.7) | | 12 (8.7) | | 0.27 | |
| TLR | 9 (2.2) | 7 (2.6) | 2 (1.4) | 0.48 | 23 (5.7) | 18 (6.7) | | 5 (3.6) | | 0.23 | |
| Stroke | 6 (1.5) | 3 (1.1) | 3 (2.2) | 0.40 | 12 (3.0) | 7 (2.6) | | 5 (3.6) | | 0.54 | |
| BARC 3 to 5 | 9 (2.2) | 5 (1.9) | 4 (2.9) | 0.50 | 10 (2.5) | 6 (2.2) | | 4 (2.9) | | 0.67 | |
| BARC 2 to 5 | 14 (3.4) | 9 (3.4) | 5 (3.6) | 0.87 | 20 (4.9) | 13 (4.9) | | 7 (5.1) | | 0.90 | |

Data are n (%). Abbreviations: BARC, Bleeding Academic Research Consortium; HBR, High Bleeding Risk; NACE, Net Adverse Clinical Events; TLF, Target-Lesion Failure; TLR, Target-Lesion Revascularization; TV-MI, Target-Vessel Myocardial Infarction. ^*^ definite or probable.

| Table S6. Clinical outcomes on- and off DAPT – troponin negative population | | | | | |  | |  | |  |
| --- | --- | --- | --- | --- | --- | --- | --- | --- | --- | --- |
|  | 0 – 3 years | On DAPT (0 – 1 month) | | | Off DAPT (1 month –3 years) | | | | | |
|  | Overall (n=889) | Non-HBR (n=621) | HBR  (n=268) | p value | Non-HBR (n=618) | | HBR  (n=267) | | p value | |
| TLF | 102 (11.5) | 21 (3.4) | 6 (2.2) | 0.36 | 51 (8.3) | | 32 (12.0) | | 0.071 | |
| NACE | 205 (23.1) | 30 (4.8) | 19 (7.1) | 0.18 | 99 (16.0) | | 69 (25.8) | | **0.001** | |
| Any death | 61 (6.9) | 3 (0.5) | 1 (0.4) | 0.82 | 24 (3.9) | | 33 (12.4) | | **<0.001** | |
| Cardiac death | 29 (3.3) | 1 (0.2) | 1 (0.4) | 0.54 | 13 (2.1) | | 14 (5.2) | | **0.010** | |
| Myocardial infarction | 54 (6.1) | 21 (3.4) | 13 (4.9) | 0.30 | 14 (2.3) | | 8 (3.0) | | 0.47 | |
| TV-MI | 28 (3.1) | 18 (2.9) | 5 (1.9) | 0.37 | 6 (1.0) | | 1 (0.4) | | 0.38 | |
| Stent thrombosis^*^ | 10 (1.1) | 1 (0.2) | 1 (0.4) | 0.54 | 4 (0.6) | | 4 (1.5) | | 0.21 | |
| Any unplanned revascularization | 105 (11.8) | 6 (1.0) | 1 (0.4) | 0.36 | 67 (10.8) | | 33 (12.4) | | 0.43 | |
| TLR | 57 (6.4) | 3 (0.5) | 0 (0.0) | 0.26 | 36 (5.8) | | 18 (6.7) | | 0.53 | |
| Stroke | 15 (1.7) | 1 (0.2) | 1 (0.4) | 0.54 | 7 (1.1) | | 6 (2.2) | | 0.18 | |
| BARC 3 to 5 | 19 (2.1) | 5 (0.8) | 3 (1.1) | 0.65 | 8 (1.3) | | 3 (1.1) | | 0.87 | |
| BARC 2 to 5 | 30 (3.4) | 6 (1.0) | 5 (1.9) | 0.27 | 11 (1.8) | | 8 (3.0) | | 0.22 | |

Data are n (%). Abbreviations: BARC, Bleeding Academic Research Consortium; DAPT, Dual Antiplatelet Therapy; HBR, High Bleeding Risk; NACE, Net Adverse Clinical Events; TLF, Target-Lesion Failure; TLR, Target-Lesion Revascularization; TV-MI, Target-Vessel Myocardial Infarction. ^*^ definite or probable.

| Table S7. Clinical outcomes on- and off DAPT – troponin positive population | | | | | |  | |  | |  |
| --- | --- | --- | --- | --- | --- | --- | --- | --- | --- | --- |
|  | 0 – 3 years | On DAPT (0 – 12 months) | | | Off DAPT (1 –3 years) | | | | | |
|  | Overall (n=599) | Non-HBR (n=461) | HBR  (n=138) | p value | Non-HBR (n=455) | | HBR  (n=127) | | p value | |
| TLF | 50 (8.3) | 19 (4.1) | 8 (5.8) | 0.39 | 16 (3.5) | | 10 (7.9) | | **0.030** | |
| NACE | 114 (19.0) | 35 (7.6) | 24 (17.4) | **0.001** | 42 (9.2) | | 20 (15.7) | | **0.026** | |
| Any death | 35 (5.8) | 4 (0.9) | 10 (7.2) | **<0.001** | 9 (2.0) | | 12 (9.4) | | **<0.001** | |
| Cardiac death | 16 (2.7) | 3 (0.7) | 4 (2.9) | **0.029** | 3 (0.7) | | 6 (4.7) | | **0.001** | |
| Myocardial infarction | 28 (4.7) | 8 (1.7) | 5 (3.6) | 0.18 | 12 (2.6) | | 6 (4.7) | | 0.20 | |
| TV-MI | 20 (3.3) | 8 (1.7) | 2 (1.4) | 0.83 | 8 (1.8) | | 3 (2.4) | | 0.62 | |
| Stent thrombosis^*^ | 6 (1.0) | 3 (0.7) | 2 (1.4) | 0.35 | 1 (0.2) | | 0 (0.0) | | 0.61 | |
| Any unplanned revascularization | 56 (9.3) | 18 (3.9) | 4 (2.9) | 0.61 | 28 (6.2) | | 8 (6.3) | | 0.85 | |
| TLR | 26 (4.3) | 11 (2.4) | 2 (1.4) | 0.53 | 12 (2.6) | | 3 (2.4) | | 0.92 | |
| Stroke | 12 (2.0) | 2 (0.4) | 3 (2.2) | **0.045** | 6 (1.3) | | 2 (1.6) | | 0.78 | |
| BARC 3 to 5 | 13 (2.2) | 7 (1.5) | 4 (2.9) | 0.28 | 2 (0.4) | | 0 (0.0) | | 0.50 | |
| BARC 2 to 5 | 22 (3.7) | 10 (2.2) | 5 (3.6) | 0.31 | 5 (1.1) | | 2 (1.6) | | 0.62 | |

Data are n (%). Abbreviations: BARC, Bleeding Academic Research Consortium; DAPT, Dual Antiplatelet Therapy; HBR, High Bleeding Risk; NACE, Net Adverse Clinical Events; TLF, Target-Lesion Failure; TLR, Target-Lesion Revascularization; TV-MI, Target-Vessel Myocardial Infarction. ^*^ definite or probable.

| Table S8. Clinical outcomes in PP-ZES vs. PF-AES HBR patients | | | | | |  |  |  |
| --- | --- | --- | --- | --- | --- | --- | --- | --- |
|  | 0 – 1 year | | | |  | 0 – 3 years | | |
|  | Overall (n=406) | PP-ZES (n=200) | PF-AES (n=206) | p value | Overall (n=406) | PP-ZES (n=200) | PF-AES (n=206) | p value |
| TLF | 25 (6.2) | 13 (6.5) | 12 (5.8) | 0.77 | 54 (13.3) | 28 (14.0) | 26 (12.6) | 0.64 |
| NACE | 67 (16.5) | 35 (17.5) | 32 (15.5) | 0.66 | 123 (30.3) | 66 (33.0) | 57 (27.7) | 0.26 |
| All-cause death | 19 (4.7) | 10 (5.0) | 9 (4.4) | 0.78 | 58 (14.3) | 29 (14.5) | 29 (14.1) | 0.88 |
| Cardiac death | 11 (2.7) | 5 (2.5) | 6 (2.9) | 0.80 | 25 (6.2) | 11 (5.5) | 14 (6.8) | 0.61 |
| Myocardial infarction | 20 (4.9) | 7 (3.5) | 13 (6.3) | 0.19 | 30 (7.4) | 12 (6.0) | 18 (8.7) | 0.30 |
| TV-MI | 7 (1.7) | 4 (2.0) | 3 (1.5) | 0.68 | 11 (2.7) | 5 (2.5) | 6 (2.9) | 0.81 |
| Stent thrombosis^*^ | 7 (1.7) | 2 (1.0) | 5 (2.4) | 0.28 | 7 (1.7) | 2 (1.0) | 5 (2.4) | 0.28 |
| Any unplanned revascularization | 18 (4.4) | 12 (6.0) | 6 (2.9) | 0.13 | 46 (11.3) | 29 (14.5) | 17 (8.3) | **0.042** |
| TLR | 9 (2.2) | 5 (2.5) | 4 (1.9) | 0.70 | 23 (5.7) | 14 (7.0) | 9 (4.4) | 0.24 |
| Stroke | 6 (1.5) | 3 (1.5) | 3 (1.5) | 0.98 | 12 (3.0) | 6 (3.0) | 6 (2.9) | 0.96 |
| BARC 3 to 5 | 9 (2.2) | 6 (3.0) | 3 (1.5) | 0.29 | 10 (2.5) | 7 (3.5) | 3 (1.5) | 0.18 |
| BARC 2 to 5 | 14 (3.4) | 10 (5.0) | 4 (1.9) | 0.090 | 20 (4.9) | 14 (7.0) | 6 (2.9) | 0.054 |

Data are n (%). Abbreviations: BARC, Bleeding Academic Research Consortium; HBR, High Bleeding Risk; NACE, Net Adverse Clinical Events; PF-AES, Polymer-Free Amphilimus-Eluting Stent; PP-ZES, Permanent Polymer Zotarolimus-Eluting Stent; TLF, Target-Lesion Failure; TLR, Target-Lesion Revascularization; TV-MI, Target-Vessel Myocardial Infarction. * definite or probable.

| Table S9. Clinical outcomes in PP-ZES vs. PF-AES non-HBR patients | | | | | |  |  |  |
| --- | --- | --- | --- | --- | --- | --- | --- | --- |
|  | 0 – 1 year | | | |  | 0 – 3 years | | |
|  | Overall (n=1082) | PP-ZES (n=541) | PF-AES (n=541) | p value | Overall (n=1082) | PP-ZES (n=541) | PF-AES (n=541) | p value |
| TLF | 63 (5.8) | 29 (5.4) | 34 (6.3) | 0.51 | 98 (9.1) | 47 (8.7) | 51 (9.4) | 0.66 |
| NACE | 109 (10.1) | 50 (9.2) | 59 (10.9) | 0.37 | 196 (18.1) | 97 (17.9) | 99 (18.3) | 0.84 |
| All-cause death | 16 (1.5) | 8 (1.5) | 8 (1.5) | 1.0 | 38 (3.5) | 19 (3.5) | 19 (3.5) | 0.99 |
| Cardiac death | 9 (0.8) | 5 (0.9) | 4 (0.7) | 0.74 | 20 (1.8) | 10 (1.8) | 10 (1.8) | 1.0 |
| Myocardial infarction | 33 (3.0) | 17 (3.1) | 16 (3.0) | 0.86 | 52 (4.8) | 31 (5.7) | 21 (3.9) | 0.16 |
| TV-MI | 28 (2.6) | 13 (2.4) | 15 (2.8) | 0.70 | 37 (3.4) | 18 (3.3) | 19 (3.5) | 0.86 |
| Stent thrombosis^*^ | 8 (0.7) | 4 (0.7) | 4 (0.7) | 1.0 | 9 (0.8) | 5 (0.9) | 4 (0.7) | 0.74 |
| Any unplanned revascularization | 54 (5.0) | 25 (4.6) | 29 (5.4) | 0.58 | 115 (10.6) | 57 (10.5) | 58 (10.7) | 0.92 |
| TLR | 33 (3.0) | 15 (2.8) | 18 (3.3) | 0.59 | 60 (5.5) | 29 (5.4) | 31 5.7) | 0.78 |
| Stroke | 6 (0.6) | 3 (0.6) | 6 (0.6) | 1.0 | 15 (1.4) | 8 (1.5) | 7 (1.3) | 0.80 |
| BARC 3 to 5 | 16 (1.5) | 7 (1.3) | 9 (1.7) | 0.61 | 22 (2.0) | 11 (2.0) | 11 (2.0) | 0.99 |
| BARC 2 to 5 | 21 (1.9) | 7 (1.3) | 14 (2.6) | 0.12 | 32 (3.0) | 13 (2.4) | 19 (3.5) | 0.27 |

Data are n (%). Abbreviations: BARC, Bleeding Academic Research Consortium; HBR, High Bleeding Risk; NACE, Net Adverse Clinical Events; PF-AES, Polymer-Free Amphilimus-Eluting Stent; PP-ZES, Permanent Polymer Zotarolimus-Eluting Stent; TLF, Target-Lesion Failure; TLR, Target-Lesion Revascularization; TV-MI, Target-Vessel Myocardial Infarction. * definite or probable.

**Figure S1**

**
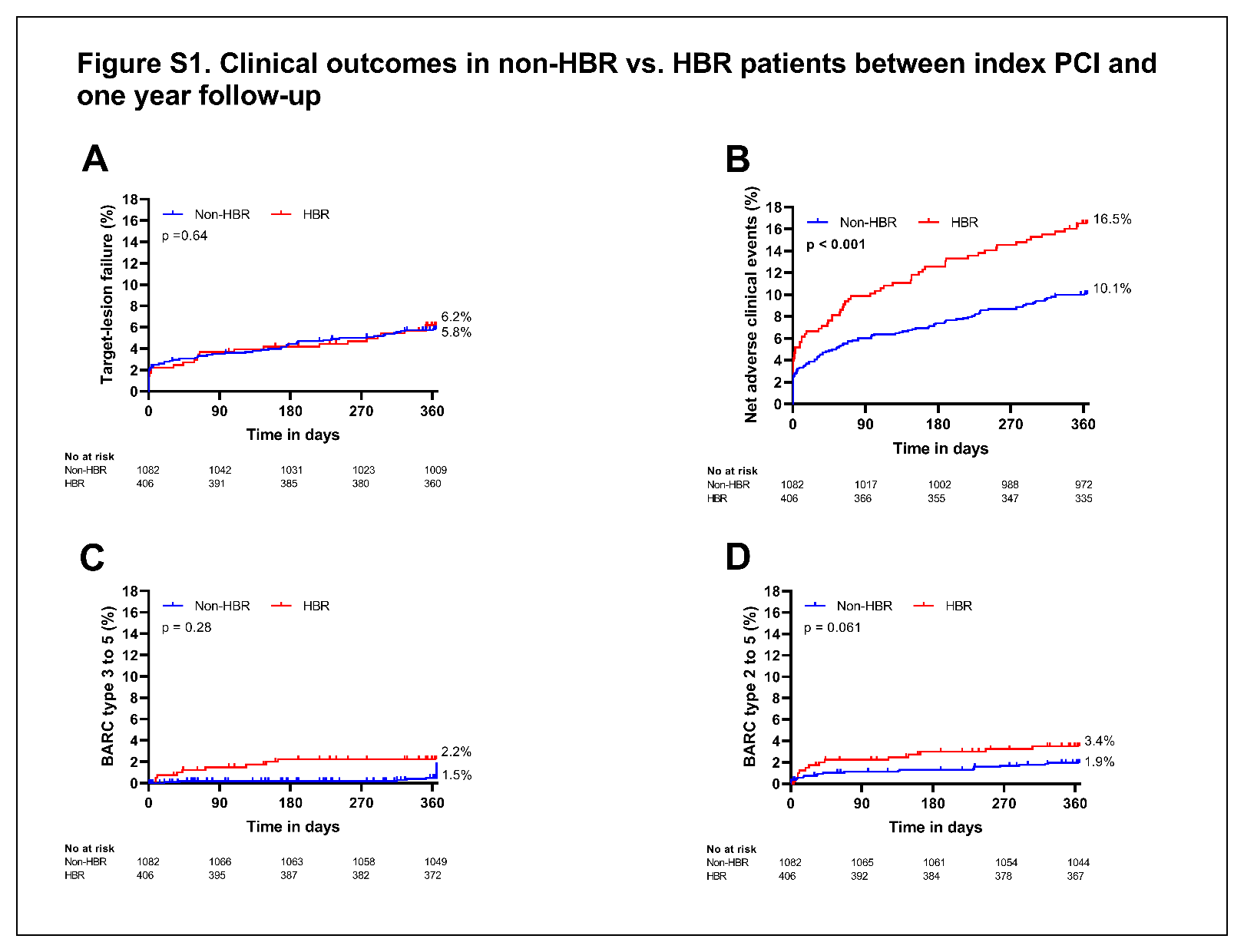
**Kaplan-Meier curves for clinical outcomes between index PCI and one year follow-up among non-HBR and HBR patients. Abbreviations: BARC, Bleeding Academic Research Consortium; HBR, High Bleeding Risk; PCI, Percutaneous Coronary Intervention. Target-lesion failure was defined as a composite of cardiac death, target-vessel myocardial infarction and target-lesion revascularisation. Net adverse clinical events was defined as a composite of all-cause death, any myocardial infarction, any unplanned revascularization, stroke and BARC 3 to 5 bleeding.


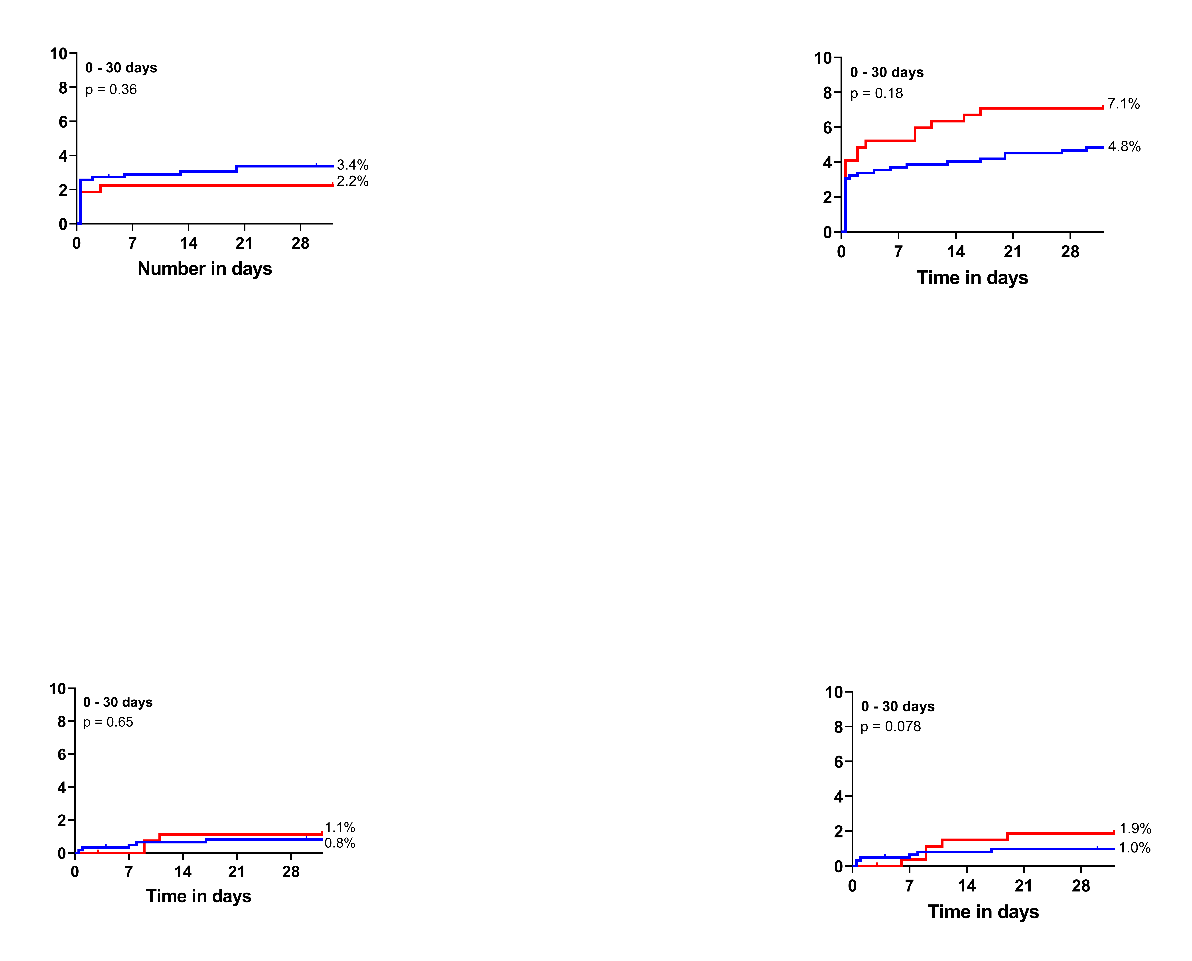
**
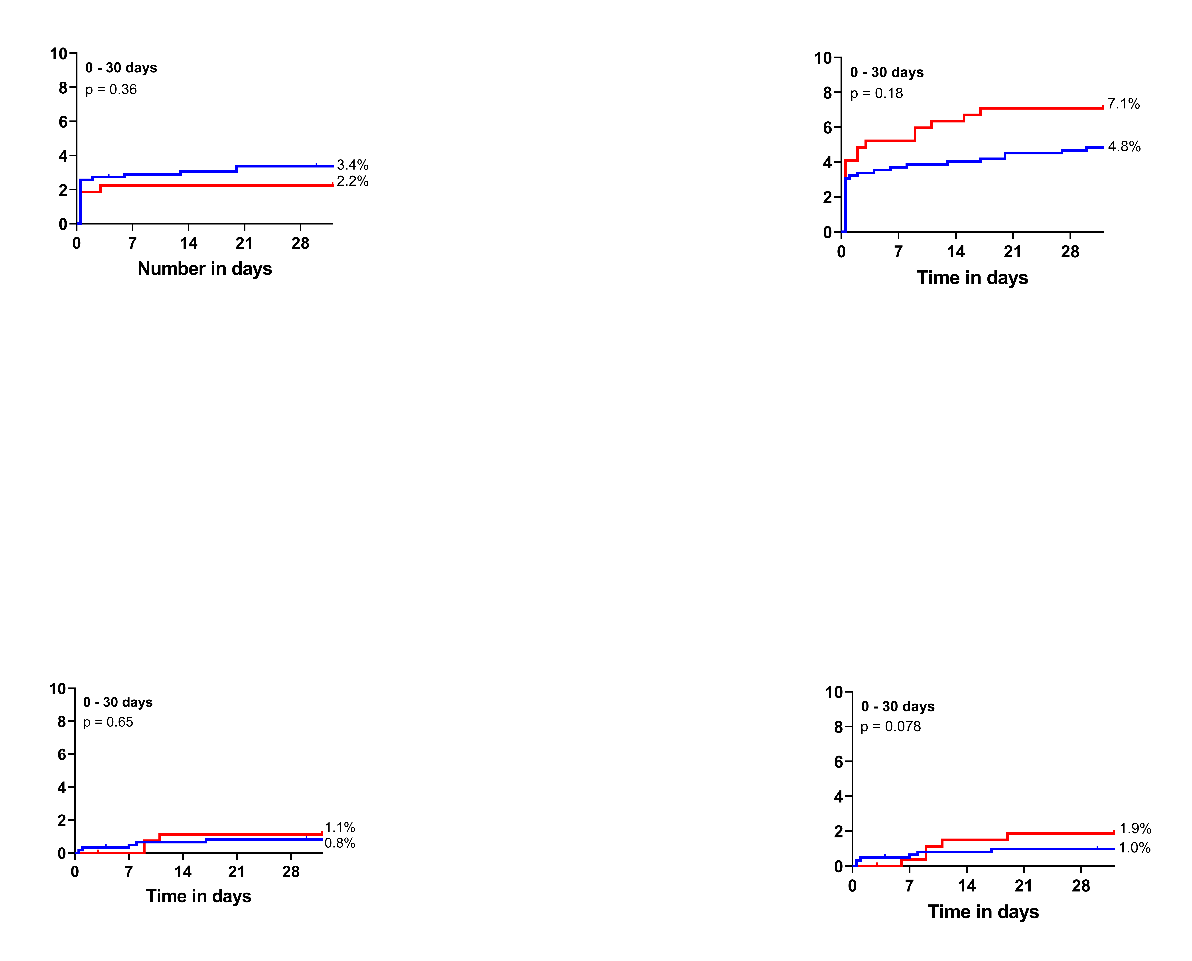
Figure S2**


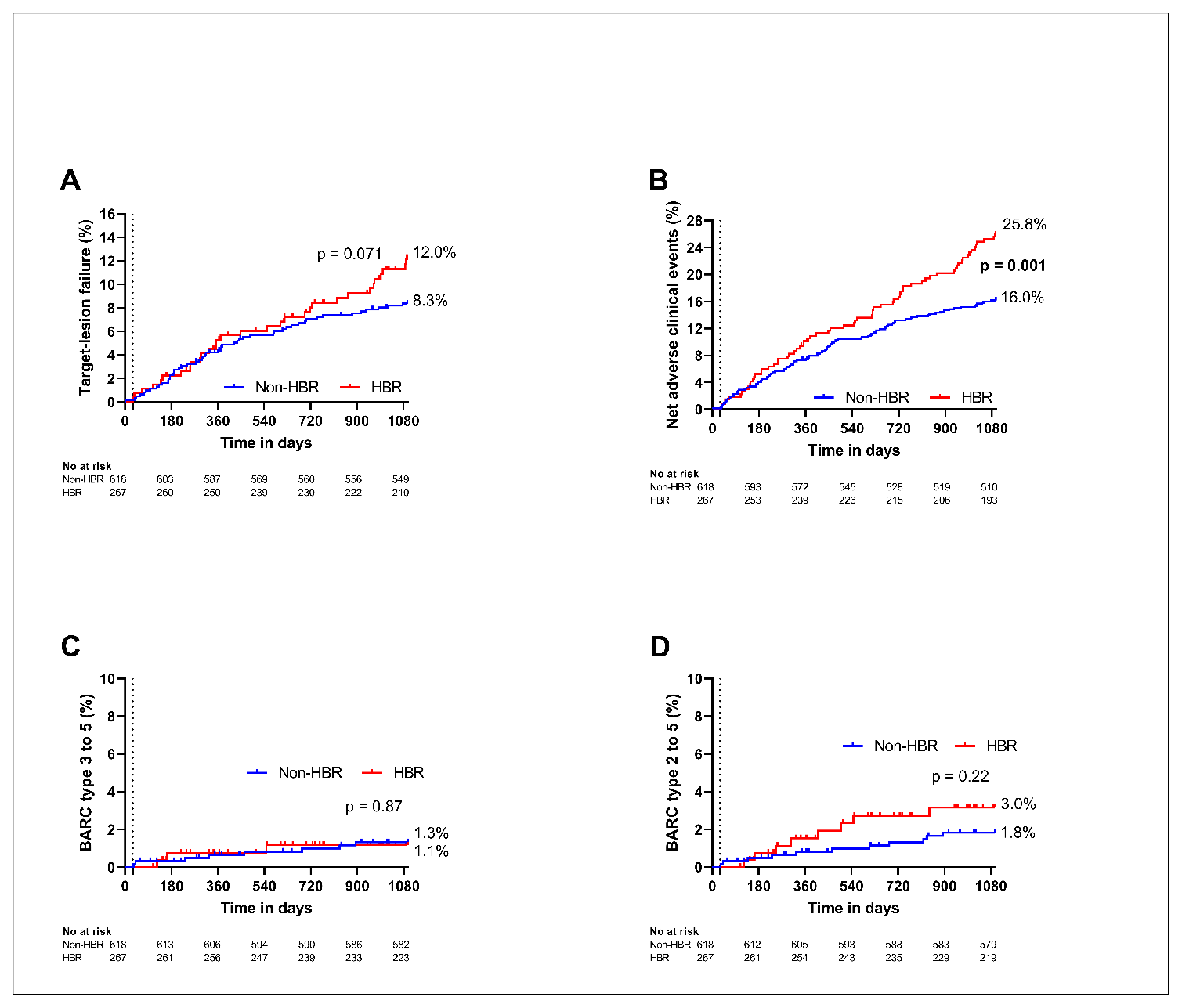


Kaplan-Meier curves for clinical outcomes in the troponin negative population in HBR vs. non-HBR patients during DAPT (inset) and after DAPT cessation. Abbreviations: BARC, Bleeding Academic Research Consortium; DAPT, Dual Antiplatelet Therapy; HBR, high bleeding risk. Target-lesion failure was defined as a composite of cardiac death, target-vessel myocardial infarction and target-lesion revascularisation. Net adverse clinical events was defined as a composite of all-cause death, any myocardial infarction, any unplanned revascularization, stroke and BARC 3 to 5 bleeding.

**Figure S3**

**
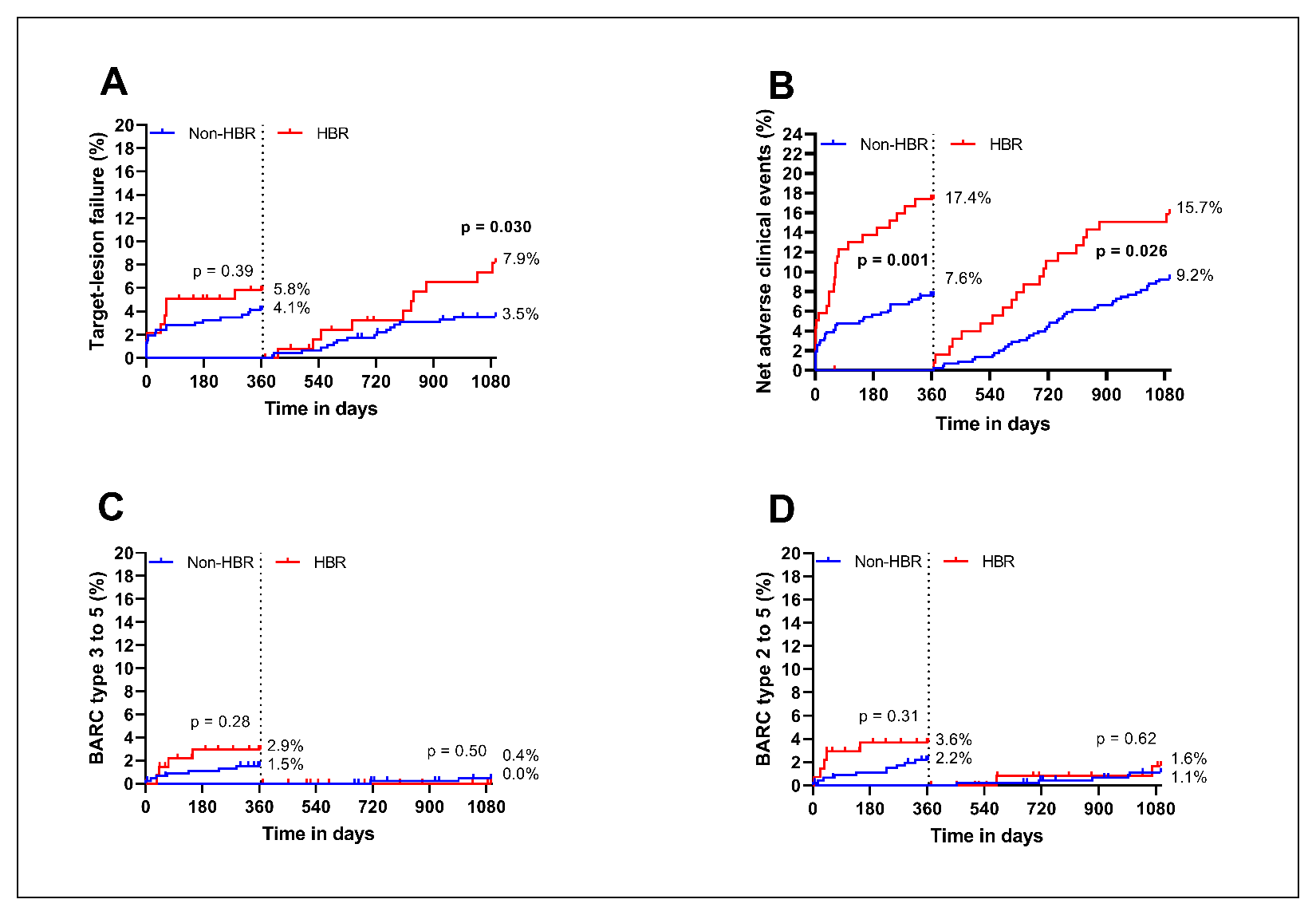
**

Kaplan-Meier curves for clinical outcomes in the troponin positive population in HBR vs. non-HBR patients during DAPT and after DAPT cessation. Abbreviations: BARC, Bleeding Academic Research Consortium; DAPT, Dual Antiplatelet Therapy. HBR, High Bleeding Risk. Target-lesion failure was defined as a composite of cardiac death, target-vessel myocardial infarction and target-lesion revascularisation. Net adverse clinical events was defined as a composite of all-cause death, any myocardial infarction, any unplanned revascularization, stroke and BARC 3 to 5 bleeding.
